# Assessing the Effectiveness and Acceptability of a Personalized Mobile Phone App in Improving Adherence to Oral Hygiene Advice in Orthodontic Patients: Protocol for a Feasibility Study and a Randomized Controlled Trial

**DOI:** 10.2196/18021

**Published:** 2021-01-13

**Authors:** Mohammad Owaise Sharif, Jonathon Timothy Newton, Susan J Cunningham

**Affiliations:** 1 Eastman Dental Institute University College London London United Kingdom; 2 King's College London London United Kingdom

**Keywords:** orthodontics, adherence, smartphone apps, mobile phone apps, personalized health care, information provision

## Abstract

**Background:**

Orthodontic treatment is a common health care intervention; treatment duration can be lengthy (2-3 years on average), and adherence to treatment advice is therefore essential for successful outcomes. It has been reported that up to 43% of patients fail to complete treatment, and there are currently no useful predictors of noncompletion. Given that the National Health Service England annual expenditure on primary-care orthodontic treatment is in excess of £200 million (US $267 million), noncompletion of treatment represents a significant inefficient use of public resources. Improving adherence to treatment is therefore essential. This necessitates behavior change, and interventions that improve adherence and are designed to elicit behavioral change must address an individual’s capability, opportunity, and motivation. Mobile phones are potentially an invaluable tool in this regard, as they are readily available and can be used in a number of ways to address an individual’s capability, opportunity, and motivation.

**Objective:**

This study will assess the effectiveness and acceptability of a personalized mobile phone app in improving adherence to orthodontic treatment advice by way of a randomized controlled trial.

**Methods:**

This study will be conducted in 2 phases at the Eastman Dental Hospital, University College London Hospitals Foundation Trust. Phase 1 is feasibility testing of the My Braces app. Participants will be asked to complete the user version of the Mobile Application Rating Scale. The app will be amended following analysis of the responses, if appropriate. Phase 2 is a randomized controlled trial to test the effectiveness and acceptability of the My Braces app.

**Results:**

This study was approved by the London – Bloomsbury Research Ethics Committee on November 5, 2019 (reference 19/LO/1555). No patients have been recruited to date. The anticipated start date for recruitment to phase 1 is October 2020.

**Conclusions:**

Given the availability, affordability, and versatility of mobile phones, it is proposed that they will aid in improving adherence to treatment advice and hence improve treatment completion rates. If effective, the applicability of this methodology to developing behavior change/modification interventions and improving adherence to treatment across health care provides an exciting opportunity.

**Trial Registration:**

ClinicalTrials.gov NCT04184739; https://clinicaltrials.gov/ct2/show/NCT04184739

**International Registered Report Identifier (IRRID):**

PRR1-10.2196/18021

## Introduction

### Overview

Orthodontic treatment is a common health care intervention; the most recent National Child Dental Health Survey (England, Wales, and Northern Ireland) indicated that 9% of 12-year-old children are receiving orthodontic treatment, and a further 37% have an unmet need [1]. Treatment duration can be lengthy (2-3 years on average), and adherence to treatment advice is therefore essential for successful outcomes [2]. Failure to adhere to treatment advice can mean that treatment objectives are not met, and there may be detrimental effects on dental health. For example, failing to adhere to dietary advice may result in breakage of the braces and increased overall treatment time, and nonadherence to dietary and oral hygiene advice during treatment can lead to avoidable tooth decay and gum disease.

The National Health Service (NHS) experiences significant costs as a result of nonadherence to treatment [3], and orthodontics is no exception to this. One multicenter study found that 43% of patients failed to complete treatment, and there were no useful predictors for this [4]. To put this into perspective, the NHS England annual expenditure on primary-care orthodontic treatment alone is in excess of £200 million (US $267 million) [5]; noncompletion of treatment therefore represents a significant inefficient use of public resources.

Improving adherence is complex and necessitates a change or modification of existing behavior. A contemporary and widely accepted framework based on multiple models of behavior change developed by Michie et al [6] is the behavior change wheel (BCW). The COM-B model forms the core of the BCW and proposes that individuals need capability (C), opportunity (O), and motivation (M) to perform or adapt a particular behavior (B).

Attempts to improve adherence to treatment advice in orthodontics have included utilizing mind maps and multimedia-assisted methods of providing information. Although these methods have been shown to significantly increase knowledge [7-9], this does not necessarily equate to better adherence to treatment advice or better outcomes [8]. A recent systematic review considered techniques including the use of motivational tests, the Hawthorne effect, and the use of awards and rewards, concluding that there is insufficient evidence to recommend a single method to improve patient adherence to treatment and that more research is required in this area [10].

Mobile phones are potentially an invaluable tool in improving adherence; they are capable of delivering both generic and personalized treatment information (for example, appointment and toothbrushing reminders) in a variety of formats (eg, visual, text, video). Ultimately, several methods for improving adherence can be delivered with a single resource. In orthodontics, mobile phones could serve to improve adherence to treatment advice, in particular adherence to oral hygiene advice, as this has been shown to be problematic in orthodontic patients. Previous research has highlighted that plaque levels in patients with fixed orthodontic appliances are 2 to 3 times higher than plaque levels in nonorthodontic patients [11]; it is crucial to optimize oral hygiene as poor oral hygiene can result in gingivitis and demineralization.

In 2019, a paper summarizing the availability of orthodontic-related apps found that 305 apps were available on the Apple App Store and Google Play Store in the United Kingdom [12]. However, to date there are very few studies assessing the effectiveness of apps for orthodontic patients. A recent systematic review that aimed to assess the effectiveness of interventions delivered by mobile phones (including apps) in improving adherence to oral hygiene advice for children and adolescents concluded that mobile phones were found to be effective [13]. However, the generalizability of these results is limited due to the small number of trials included (n=2) and the unclear risk of bias of the included studies [13]. Additionally, none of the included studies assessed the effectiveness of personalized information provision, despite evidence that personalized communication in health care is more effective than nonpersonalized communication in relation to changing health-related behaviors [14]. More recently, a pilot study demonstrated the potential effectiveness of a chat room via a mobile phone app in improving appointment attendance and reducing relapse in patients wearing retaining braces [15]. Furthermore, a randomized controlled trial assessing the effectiveness of a personalized mobile phone app in supporting orthodontic patients during fixed appliance treatment demonstrated the potential for improvement in oral hygiene at 3 months into treatment, with the app group demonstrating a greater reduction in gingival bleeding and plaque scores [16].

To date, there have been no randomized controlled trials published in orthodontics assessing the effectiveness of behavior change interventions designed using the BCW. In light of this, an app called “My Braces”, which is grounded in behavior change theory, has been developed by the research team in order to provide generic and personalized orthodontic treatment information. The personalized element allows patients to input their own treatment information (including progress photographs), set goals, and develop plans for achieving these goals and provides the patient and clinicians with appropriate dashboards to monitor progress. Ultimately, this app aims to improve adherence to orthodontic treatment advice. For the purposes of this study, the My Braces app has been designed to allow three different levels of access and functionality:

Version 1 includes a toothbrushing timer only.

Version 2 includes a toothbrushing timer and generic treatment information (a combination of images and text).

Version 3 includes a toothbrushing timer; generic treatment information; access to input patient-specific personalized treatment information (including progress photographs), set goals, and develop plans for achieving these goals; and appropriate dashboards for the patient and clinicians to monitor progress.

The longitudinal nature of orthodontic treatment makes this an ideal opportunity to assess the effectiveness of new ways to provide information in order to improve adherence to treatment advice. If found effective, the applicability of this methodology to improve adherence across health care provides an exciting opportunity.

### Aims

This study will assess the quality, effectiveness, and acceptability of the My Braces app and test the following primary hypothesis:

Using the full functionality of the My Braces app improves adherence to oral hygiene advice in patients undertaking fixed brace treatment.

The secondary hypothesis is as follows:

Using the full functionality of the My Braces app improves adherence to orthodontic treatment advice (reduction in fixed appliance breakages, missed and rescheduled appointments, treatment duration, number of appointments, and enamel demineralization).

## Methods

This study will be conducted in 2 phases at the Eastman Dental Hospital (EDH), University College London Hospitals (UCLH) Foundation Trust, London, United Kingdom.

### Consent Process

A member of the direct care team (the clinician undertaking treatment) will ask eligible patients if they would be prepared to consider involvement in the study. Children younger than 16 years will only be approached if accompanied by a parent/legal guardian. All prospective participants will be provided with patient information leaflets and will be given ample opportunity to ask questions about the study before enrolling. Participants will be required to provide written informed consent to participate in the study. For participants younger than 16 years, parents/legal guardians will also be required to provide written informed consent for their child to participate in this study. Contact details for a designated member of the research team will be provided, and patients will be informed that they may withdraw from the study at any point if they wish to do so. If this happens and participants give a reason for doing so, that reason will be recorded. Withdrawing from the trial will not affect treatment in any way. Signed consent and assent for inclusion will be obtained by a member of the research team. All members of the team have undertaken training in research ethics, including completion of Good Clinical Practice in Secondary Care. Example consent forms can be obtained by contacting the corresponding author.

### Phase 1

Phase 1 is a feasibility study with a convenience sample of patients at different stages of treatment. The purpose of this phase is to assess whether the app is deemed to be of high quality by end users.

#### Inclusion Criteria

The study will include patients already accepted for fixed orthodontic appliance treatment (“train track”–type braces) at the EDH. Patients at all stages of treatment will be recruited. Patients must also be 10-18 years of age (inclusive), be familiar with and have daily access to a smartphone or tablet, and be able to read and communicate in English, as the app is currently available only in English.

#### Exclusion Criteria

Patients with severe hypodontia (developmental absence of teeth) and craniofacial/orthognathic patients will be excluded from this study. These patients require complex multidisciplinary treatment; the information needs for these patients are therefore different. Treatment involves input from specialties other than orthodontics, and this may therefore influence adherence to treatment advice.

Patients with communication difficulties (for example, severe autism or learning difficulties) will also be excluded from the study.

#### Recruitment

A convenience sample will be recruited for phase 1. Patients meeting the inclusion criteria and willing to participate in the feasibility study will be enrolled. Patients at different stages of treatment will be recruited as follows: 20 patients who have been accepted for fixed orthodontic appliance treatment but who have not yet started treatment, 20 patients who are currently in fixed appliance treatment, and 20 patients who have completed treatment and are wearing retainers.

The reason for allocating patients in this manner is that the app is designed to support the whole treatment journey (before, during, and after active treatment). Recruiting patients at different stages of this journey therefore ensures that all relevant components of the app are experienced and assessed by an appropriate patient group.

#### Data Collection

The app will be assessed by current orthodontic patients and tested for quality using the user version of the Mobile Application Rating Scale (uMARS) [17]. The uMARS is a reliable tool for assessing app quality and consists of sections relating to app engagement, functionality, aesthetics, and information [15]. The uMARS quality score can range from 1 (“Inadequate”) to 5 (“Excellent”). Additionally, there is a free text section for patients to provide comments.

It is estimated that the total duration of this phase for each participant will be around 8-12 weeks. Patients will be provided with access to the app and asked to complete the uMARS questionnaire prior to, or at, the next appointment. Patients will be prompted by the My Braces app to complete the uMARS form online using UCL Opinio. After this point, no further data will be collected.

The following baseline details will also be obtained: age, gender, and stage of treatment.

#### Planned Analyses

The Kruskal-Wallis test will be used to compare the uMARS scores across the three groups and the scores across different sections of the uMARS form.

Any changes felt to be appropriate based on feedback from the uMARS questionnaire will be implemented in the app prior to using it in phase 2.

### Phase 2

#### Overview

Phase 2 is a randomized controlled trial. The purpose of this phase is to assess the effectiveness of the My Braces app in improving adherence to oral hygiene advice in orthodontic patients. Table 1 is the schedule of enrolment and assessments.

**Table 1:** Schedule of enrolment and assessments (groups A, B, and C will receive different versions of the intervention, the My Braces app).

Characteristic		Screening	Intervention phase		Final visit
		Visit 1	Visit 2	Visit 3	Visit 4
Visit timing		Baseline	3 months	12 months	End of treatment
Window of flexibility for timing of visits		N/A^a^	1 month	2 months	1 month
**Assessment**					
	Informed consent	✓	N/A	N/A	N/A
	Medical history	✓	✓	✓	✓
	Eligibility confirmation	✓	✓	N/A	N/A
	Taking photographs	✓	N/A	N/A	✓
	Type of dental bite and whether teeth were removed	✓	N/A	N/A	N/A
	Randomization	✓	N/A	N/A	N/A
	Bleeding scores	✓	✓	✓	✓
	Plaque scores	✓	✓	✓	✓
	App subjective quality assessment	N/A	✓	N/A	✓
	Adverse events review	✓	✓	✓	✓

^a^N/A: not applicable.

#### Inclusion Criteria

The study will include patients who have been accepted for fixed orthodontic appliance treatment (“train track”–type braces) at the UCLH NHS Foundation Trust EDH but who have not yet commenced treatment. Patients must also be 10-18 years of age (inclusive) at the start of treatment, have a sufficient number of permanent teeth erupted to allow outcome assessment, be familiar with and have daily access to a smartphone or tablet, and be able to read and communicate in English.

#### Exclusion Criteria

Patients with severe hypodontia (developmental absence of teeth) and craniofacial/orthognathic patients will be excluded from this study. These patients require complex multidisciplinary treatment; the information needs for these patients are therefore different, and treatment involves input from specialties other than orthodontics, and this may therefore influence adherence to treatment advice.

Patients with communication difficulties (for example, severe autism or learning difficulties) will also be excluded from the study.

Patients who are aware that a family member or friend is enrolled in the study (phase 1 or phase 2) will be excluded from the study because it is possible that these patients may become aware of details of the app that may not be relevant to the group that they are randomized to.

#### Recruitment

It is anticipated that recruitment will take approximately 18 months. Patients meeting the inclusion criteria and willing to participate in the trial will be randomized to one of the three study groups:

Group A will act as the control group and will be provided with standard treatment information (verbal and written) and access to the basic version 1 of the app with just a toothbrushing timer. The timer is necessary as the health behavior outcome is toothbrushing duration.

Group B will be provided with standard treatment information (verbal and written) and access to version 2 of the app, which has a toothbrushing timer and provides generic treatment information (a combination of images and text).

Group C will be provided with standard treatment information (verbal and written), and patients will have access to version 3 of the app; this group will have access to the full functionality of the app, which will allow patients to input their own personalized treatment information (including progress photographs), set goals, and develop plans for achieving these goals and will provide the patient and clinicians with appropriate dashboards to monitor progress.

It is important to include Group B, as this will allow the team to ascertain whether it is the personalization of the app content that confers any benefit over and above providing generic information via an app if a difference is found between Groups A and C.

#### Randomization and Allocation Concealment

Randomization will be undertaken using computer-generated random number sequences; block randomization (blocks of 6) will be used to help ensure balance in the allocation of participants to each arm of the trial. Sealed opaque envelopes will be used to ensure allocation concealment. This aspect of the study will be carried out by a member of the research team not involved in recruitment/data collection; this team member will also hold the randomization list and maintain a trial subject enrolment log.

#### Data Collection

A complete data set will be collected at baseline, 3 months into treatment, 12 months into treatment, and at the end of treatment when the orthodontic appliances have been removed.

Two primary outcome measures have been selected, a health behavior outcome and a clinical outcome, as both are felt to be important. The outcome measures are timed toothbrush use and gingival bleeding scores (these scores indicate the amount of bleeding from the gums, where bleeding is an indicator of inadequate toothbrushing).

The secondary outcomes of interest are as follows: plaque scores, which indicate the amount of plaque on the teeth, where retention of plaque is an indicator of inadequate toothbrushing; number of fixed appliance breakages; number of missed and rescheduled appointments; treatment duration, number of appointments (including emergency appointments for appliance breakages); enamel demineralization (early tooth decay) on the upper anterior six teeth (using standardized photographs), as demineralization is a further indicator of inadequate toothbrushing and nonadherence to the appropriate diet; subjective quality of the app (sections E and F of the uMARS questionnaire); and app engagement.

The following baseline measurements and details will be obtained prior to treatment commencing: age, gender, malocclusion (type of dental bite), whether or not extractions (removal of teeth) are required for treatment, clinical photographs of the teeth (these will be compared with completion of treatment photographs to assess for enamel demineralization), bleeding score, and plaque score.

#### Methods of Outcome Assessment

##### Timed Toothbrush Use

Timed toothbrush use will be determined via the information stored on the app. It is anticipated that participants will not remember to time every brushing episode; therefore, the duration and number of brushing sessions stored within the app will allow for the average brushing duration to be determined. The unit of measurement will be seconds.

##### Gingival (Gum) Bleeding

A periodontal probe (specialized probe to test gum health) will be used; the presence of bleeding within 10 to 30 seconds of probing is indicative of inflammation of the gingivae (gums). Scores will be recorded for all teeth anterior to the molars (incisors, canines, and premolars) and will be recorded as the percentage of the total number of tooth surfaces (6 surfaces per tooth). A binary score will be assigned to each surface (1 for bleeding present, 0 for no bleeding).

A calibration exercise will be conducted prior to commencing the study. The key factors affecting consistency of probing technique are probe position and pressure applied (ideally 20 to 25 grams). The bleeding assessment technique will therefore be reviewed and observed in a volunteer subject by a gold standard examiner following training performed on models. It is not possible to do duplicate bleeding assessments as the act of repeated probing can cause bleeding; therefore, it is essential that this aspect of the study is well controlled for.

##### Plaque Scores

Plaque scores will be recorded for all teeth anterior to the molars (incisors, canines, and premolars) as the percentage of total surfaces (6 surfaces per tooth). A plaque disclosing agent (a temporary dye that is retained on the plaque to make it visible) will be used for the plaque assessment. A binary score will be assigned to each surface (1 for plaque present, 0 for no plaque present) [18].

Prior to the initiation of the study, 10 non–study subjects with similar characteristics to those who will be included in the study will be recruited for an examiner calibration exercise. A request for volunteers will be made from current patients within the orthodontic department. The designated examiner (MOS) will measure full mouth plaque scores in the manner described for all 10. This will be repeated on the same day (at least 15 minutes apart to reduce memory bias); the examiner will evaluate the same subjects for a second time with no brushing undertaken in between. Subjects will be redisclosed for the second plaque scoring.

Intra-examiner repeatability for plaque measurement will then be assessed.

##### Enamel Demineralization

Before placing the orthodontic braces, teeth will be polished with a rubber cup and fluoride-free pumice paste and dried with air, and standardized digital photos will be taken under identical lighting conditions and with the same camera settings. These will be used to assess for enamel demineralization [19]. Three photographs will be obtained (front, right, and left with the teeth biting together) and stored securely.

At the end of treatment, the brace and the adhesive used to secure it to the teeth will be removed, and the teeth will be polished with a rubber cup and fluoride-free pumice paste and dried with air. A further series of standardized photographs will be obtained.

For the evaluation of demineralization, photographs will be assessed, and the severity of enamel demineralization will be recorded. The labial surfaces of the upper incisors and canines will be scored as follows [20]: (1) no white spot formation, (2) slight white spot formation (thin rim), (3) excessive white spot formation (thicker bands), or (4) white spot formation with cavitation.

A random sample of photographs will be re-examined after 1 month to check reliability.

These photographs are an essential part of routine orthodontic records for all patients, and therefore no additional patient appointments or increase in appointment time will be needed.

##### Other Outcomes

Information relating to treatment duration and the number of breakages (for example, detachment of the brace from the tooth, displacement of the brace wire), missed or rescheduled appointments, and emergency appointments will be obtained from the patient’s medical records. Data relating to the subjective quality score for the app will be obtained by way of patient-completed paper questionnaires (sections E and F of the uMARS form). Furthermore, app engagement will be determined via the data available on Google’s Firebase system.

#### Blinding

The outcome assessor will be blinded; however, given the nature of the intervention, it is not possible to blind the patient.

#### Sample Size

The following assumptions have been made in the sample size calculation:

Data are normally distributed and initial comparison of the 3 groups would use an analysis of variance (ANOVA), followed by 2-sample *t* tests for A versus B, B versus C, and A versus C if any statistical significance is found.

The significance level is *P*<.05 with 80% power.

##### Timed Toothbrush Use

Using a clinically relevant difference of 20 seconds between groups and a SD of 23.6 seconds from a paper by Tesini and Perlman [21] (standardized difference=0.85) and accounting for multiple testing, 29 patients are required in each group (n=87). Assuming a dropout of 20%, the sample size is 35 per group.

##### Gingival Bleeding

An estimated clinically relevant difference of 15% and a SD of 20% (standardized difference=0.75) was used, based on advice from Dr Jeanie Suvan (clinical trials coordinator, Restorative Dental Sciences, UCL Eastman Dental Institute, Faculty of Medical Sciences, UCL), who has experience in commercial trials using these outcome measures. The same assumptions were followed as described above, and this indicated a sample size of 38 per group. With the 20% dropout factored in, the sample size calculation is 46 per group (n=138).

Therefore, 138 patients will be recruited. There are a number of assumptions in these calculations; therefore, an internal pilot will be conducted, and the calculations will be repeated using 3-month data for the first 15 patients recruited to each group.

#### Planned Analyses

Proposed outcome analyses are summarized in Table 2.

For each of the main outcome measures, it is anticipated that regression analysis will be undertaken. The main factor of interest (group allocation) and confounding variables (eg, age, gender, ethnicity) will be entered into univariable regression analyses to determine the level of significance and to limit the number of variables entered into the multivariable analysis. Any factors with *P*≤.10 will be considered for inclusion in the multivariable analysis. Factors with *P*<.05 in the multivariable analysis will be considered as having a significant effect on toothbrushing time or gingival bleeding. Secondary outcomes will be explored in a similar manner.

If necessary, an intention to treat analysis will be performed; all enrolled and randomized patients will be included in the analysis and analyzed in the groups to which they were randomized.

**Table 2:** A summary of proposed statistics for outcome analysis.

Outcomes	Method of analysis
Toothbrushing duration	Comparison of the 3 groups using an ANOVA, followed by 2-sample *t* tests (A vs B, B vs C, and A vs C) if any statistical significance is found
Bleeding score	Chi-square test
Plaque scores	Comparison of the 3 groups using an ANOVA, followed by 2-sample *t* tests (A vs B, B vs C, and A vs C) if any statistical significance is found
Number of breakages	Kruskal-Wallis test, followed by Mann-Whitney test (A vs B, B vs C, and A vs C) if any statistical significance is found
Number of missed appointments	Kruskal-Wallis test, followed by Mann-Whitney test (A vs B, B vs C, and A vs C) if any statistical significance is found
Total number of appointments	Kruskal-Wallis test, followed by Mann-Whitney test (A vs B, B vs C, and A vs C) if any statistical significance is found
Treatment duration	Comparison of the 3 groups using an ANOVA, followed by 2-sample *t* tests (A vs B, B vs C, and A vs C) if any statistical significance is found
Enamel demineralization	Chi-square test
App subjective quality	Comparison of the 3 groups and different section of the uMARS form by using the Kruskal-Wallis test.
App engagement	Comparison of the 3 groups using an ANOVA, followed by 2-sample *t* tests (A vs B, B vs C, and A vs C) if any statistical significance is found

### Data Handling and Confidentiality

For phase 1, all responses to the uMARS questionnaire will be received anonymously. For phase 2, the researchers will receive a complete list of the random user log-in codes (randomly generated using letters and numbers only) from the app developers; these log-in codes will divide participants into the three groups (A, B, and C). These codes will be used to randomly assign patients. Details relating to toothbrushing duration and app engagement will be logged against these codes; however, it will not be possible to identify individuals from these codes alone. A separate document linking the log-in codes to an individual’s unique trial ID will be held on secure storage devices (mobile devices will have Advanced Encryption Standard 256-bit encryption, which has been made a security standard within the NHS). Only members of the research team or those regulatory bodies that are involved in monitoring research studies will have access to this data, and it will only be used for the purposes of analysis or if unblinding is required.

The app has been developed in such a manner that the researchers will not be able to access progress photographs (these will be stored on the patients’ camera rolls), location data, other apps on the patients’ mobile devices, or contacts.

### Study Monitoring

Given that there is no morbidity or mortality risk, an independent data monitoring committee will not be established. However, an interim data analysis will be conducted using 3-month data for the first 15 participants in each group, and the research team will meet to review these and take any action if necessary.

The research team will meet on a regular basis to ensure the research is progressing with no difficulties; this will include reviewing of recruitment rates. It is intended that the Research Ethics Committee and study sponsor will be updated if a substantial amendment is made to the conduct of this research.

## Results

This study was approved by the London – Bloomsbury Research Ethics Committee on November 5, 2019 (reference 19/LO/1555). No patients have been recruited to date. The anticipated start date for recruitment is October 2020.

## Discussion

Improving adherence to treatment advice in orthodontics necessitates a change/modification of existing behaviors. High noncompletion rates [4] and poor plaque control among orthodontic patients [11] support the notion that nonadherence to treatment advice is a significant issue for orthodontic patients. Given the availability, affordability, and versatility of mobile phones, it is proposed that using this technology will aid in improving adherence to treatment advice and hence treatment completion. Furthermore, several methods for improving adherence could be delivered within a single app, and the experience can be made personal for each patient. In this study, the effectiveness of a personalized mobile phone app in improving adherence to orthodontic treatment advice will be investigated.

If effective, the applicability of this methodology to developing behavior change/modification interventions and improving adherence to treatment across health care provides an exciting opportunity.

## Abbreviations

ANOVA: analysis of variance
BCW: behavior change wheel
EDH: Eastman Dental Hospital
NHS: National Health Service
SPIRIT: Standard Protocol Items: Recommendations for Interventional Trials
UCLH: University College London Hospitals
uMARS: user version of the Mobile Application Rating Scale

## References

[1] Rolland SL, Treasure E, Burden DJ, Fuller E, Vernazza CR (2016). The orthodontic condition of children in England, Wales and Northern Ireland 2013. Br Dent J.

[2] Fleming PS, Scott P, DiBiase AT (2007). Compliance: getting the most from your orthodontic patients. Dent Update.

[3] Howe E (2011). Action on medicine wastage and improving medicine use.

[4] Mandall NA, Matthew S, Fox D, Wright J, Conboy FM, O'Brien KD (2008). Prediction of compliance and completion of orthodontic treatment: are quality of life measures important?. Eur J Orthod.

[5] NHS England, Chief Dental Officer team (2015). Guide for Commissioning Orthodontics.

[6] Michie S, van SMM, West R (2011). The behaviour change wheel: a new method for characterising and designing behaviour change interventions. Implement Sci.

[7] Thickett E, Newton JT (2006). Using written material to support recall of orthodontic information: a comparison of three methods. Angle Orthod.

[8] Patel JH, Moles DR, Cunningham SJ (2008). Factors affecting information retention in orthodontic patients. Am J Orthod Dentofacial Orthop.

[9] Srai JPK, Petrie A, Ryan FS, Cunningham SJ (2013). Assessment of the effect of combined multimedia and verbal information vs verbal information alone on anxiety levels before bond-up in adolescent orthodontic patients: a single-center randomized controlled trial. Am J Orthod Dentofacial Orthop.

[10] Aljabaa A, McDonald F, Newton JT (2015). A systematic review of randomized controlled trials of interventions to improve adherence among orthodontic patients aged 12 to 18. Angle Orthod.

[11] Klukowska M, Bader A, Erbe C, Bellamy P, White DJ, Anastasia MK, Wehrbein H (2011). Plaque levels of patients with fixed orthodontic appliances measured by digital plaque image analysis. Am J Orthod Dentofacial Orthop.

[12] Siddiqui NR, Hodges S, Sharif MO (2019). Availability of orthodontic smartphone apps. J Orthod.

[13] Sharif MO, Newton T, Cunningham SJ (2019). A systematic review to assess interventions delivered by mobile phones in improving adherence to oral hygiene advice for children and adolescents. Br Dent J.

[14] Kreuter MW, Holt CL (2016). How Do People Process Health Information? Applications in an Age of Individualized Communication. Curr Dir Psychol Sci.

[15] Zotti Francesca, Zotti Rinaldo, Albanese Massimo, Nocini Pier Francesco, Paganelli Corrado (2019). Implementing post-orthodontic compliance among adolescents wearing removable retainers through Whatsapp: a pilot study. Patient Prefer Adherence.

[16] Scheerman JFM, van Meijel B, van Empelen P, Verrips GHW, van Loveren C, Twisk JWR, Pakpour AH, van den Braak MCT, Kramer GJC (2020). The effect of using a mobile application ("WhiteTeeth") on improving oral hygiene: A randomized controlled trial. Int J Dent Hyg.

[17] Stoyanov SR, Hides L, Kavanagh DJ, Wilson H (2016). Development and Validation of the User Version of the Mobile Application Rating Scale (uMARS). JMIR Mhealth Uhealth.

[18] O'Leary TJ, Drake RB, Naylor JE (1972). The plaque control record. J Periodontol.

[19] Stecksén-Blicks C, Renfors G, Oscarson ND, Bergstrand F, Twetman S (2007). Caries-preventive effectiveness of a fluoride varnish: a randomized controlled trial in adolescents with fixed orthodontic appliances. Caries Res.

[20] Gorelick L, Geiger AM, Gwinnett A (1982). Incidence of white spot formation after bonding and banding. Am J Orthod.

[21] Tesini D A, Perlman S P (1994). The effect of a color-changing toothbrush with and without instruction on the duration of toothbrushing. Pediatr Dent.

